# Drug-Loaded Lipid-Core Micelles in Mucoadhesive Films as a Novel Dosage Form for Buccal Administration of Poorly Water-Soluble and Biological Drugs

**DOI:** 10.3390/pharmaceutics12121168

**Published:** 2020-11-30

**Authors:** Wai-Houng Chou, Ariel Galaz, Miguel O. Jara, Alexander Gamboa, Javier O. Morales

**Affiliations:** 1 Department of Pharmaceutical Science and Technology, School of Chemical and Pharmaceutical Sciences, University of Chile, Santiago 8380494, Chile; wai-houng.chou@postqyf.uchile.cl (W.-H.C.); ariel.galaz@ug.uchile.cl (A.G.); miguel.jara@utexas.edu (M.O.J.); agamboa@ciq.uchile.cl (A.G.); 2 Advanced Center for Chronic Diseases, Santiago 8380494, Chile; 3 Center of New Drugs for Hypertension, Santiago 8380494, Chile; 4 Division of Molecular Pharmaceutics and Drug Delivery, College of Pharmacy, University of Texas at Austin, Austin, TX 78712, USA

**Keywords:** lipid-core micelles, rhodamine 123, poorly water-soluble drugs, human insulin, biological drugs, mechanical properties, mucoadhesive films, buccal administration, in vitro release, ex vivo permeation

## Abstract

The aim of the study was to develop a novel buccal dosage form to transport rhodamine 123 and human insulin as models for poorly water-soluble and biological drugs, using lipid-core micelles (LCMs)-loaded mucoadhesive films. LCMs were synthesized by a low-energy hot emulsification process, yielding spherically shaped, small-sized, monodispersed and negatively charged carriers with high entrapment efficiency. In vitro release studies demonstrated a higher release of insulin rather than rhodamine from LCMs in simulated physiological conditions, due to an initial burst release effect; however, both release profiles are mainly explained by a diffusion mechanism. Furthermore, LCMs-loaded mucoadhesive films were manufactured and preserved with similar mechanical properties and optimal mucoadhesive behavior compared to nonloaded films. Ex vivo permeation experiments using excised porcine buccal epithelium reveal that both rhodamine and insulin-loaded LCM films elicited a significantly enhanced permeation effect compared to LCMs in suspension and free drugs in solution as controls. Hence, LCMs-loaded mucoadhesive films are suitable as buccal dosage form for the transport and delivery of rhodamine 123 and insulin, as models for poorly water-soluble and biological drugs, respectively.

## 1. Introduction

Among enteral routes for drug administration, the oral is the most used, comfortable and tolerated by patients. Nonetheless, several disadvantages are reported, including fluctuation of pH values between gastric and intestinal segments, presence of lytic enzymes, epithelial barriers surrounding the intestinal absorptive lumen, gastric emptying time and intestinal motility, presence of food, presystemic first pass metabolism and elimination, and the administration of irritating or unstable to gastric conditions drugs [1]. These issues make it difficult for the administration of poorly water-soluble, as well as biological drugs, and therefore, lead to nonpredictable pharmacokinetic and pharmacodynamic profiles [2].

Overcoming those limiting factors, the buccal administration has emerged as an alternative for the oral route and attracted the interest of research groups [3,4]. The buccal mucosa is accessed through the inner lining of cheeks and represents a mucosal drug administration route. The buccal epithelium is constituted by 40 to 50 layers of nonkeratinized, squamous and stratified epithelial cells, creating a 100 to 800 μm thickness barrier upon highly vascularized submucosal layer [5,6]. One of the main benefits of this administration is the feasibility to administer controlled-drug delivery systems, due to the slow rate of tissue replacement and salivary wash, and less shear effect related to the masticatory process [7]. Moreover, the presence of negatively charged glycoproteins and main constituents of saliva, the so-called mucins, have shown to promote the adhesion to the mucus (mucoadhesion) of certain delivery systems, favoring the interaction between drugs and the absorptive layer, leading to an enhanced permeation profile [6]. In that sense, several types of buccal dosage formulations have been approved by the United States Food and Drug Administration (FDA), including buccal tablets of fentanyl, miconazole and acyclovir; mucoadhesive patches and films of lidocaine, fentanyl, buprenorphine alone or combined with naloxone, and diazepam; and chewing gum for nicotine [8]. Furthermore, several investigations have been conducted to develop other approaches using different technologies and dosage forms, such as orodispersable wafers [9], sponges [10], mucoadhesive hydrogels [11], mucoadhesive films based on graft copolymers [12], spray [13] and nanoparticles [14]. When designing nanoparticles as dosage forms for buccal administration, their physical–chemical properties need to be considered, to ensure an optimal interaction with the mucosal barrier [15]. Besides, formulations containing nanoparticles should lead to stable dosage forms during manufacturing and storage conditions, and must increase the residence time in the buccal epithelium to enhance drug permeability, and thus, improve absorption [15]. Buccal film formulations have been proposed to achieve all of these requirements. Films are referred to as thin and flexible layers of polymer that may include drugs and other systems, eliciting several advantages compared to other buccal dosage forms, including enhanced bioavailability, better patient compliance, noninvasiveness and ease to handle during manufacturing and transport [16]. Furthermore, the type of polymer chosen for the elaboration of buccal films may influence in several pharmacokinetic parameters of drug absorption and controlled the release. Mucoadhesive polymers can be used to bind to mucin in the mucus layer, favoring the interactions of the dosage form and the absorptive mucus layer of the cheeks, and thus, increasing the contact time between films and the mucus and enhancing the absorption process [6]. Considering the mentioned advantages, there are several candidates using films to deliver drugs that are currently facing various stages of clinical trials, with promising results that may lead to marketed products in the near future [15].

Lipid-based nanoparticles have been reported to be more advantageous in comparison with other types of particles, due to a less toxic profile for in vivo applications, drug loading efficiency and controlled drug release potential [17]. Lipid-core micelles (LCMs) are classified as polymer-lipid hybrid particles and are constituted by self-assembled amphiphilic copolymers [18]. The copolymers are made by lipid constituents (e.g., fatty acids or glycerides) coupled with a more hydrophilic portion, usually containing poly(ethylene glycol) (PEG) units. Therefore, in contact with water, these copolymers tend to assemble by forming a stable lipid core surrounded by the hydrophilic segment, which is in direct contact with the aqueous medium [18]. Several advantages are attributed to LCMs, including a particle size ranging from 5 to 100 nm, entrapment of poorly water-soluble drugs, enhanced permeation through biological barriers, controlled release of trapped drugs, feasibility to conjugate with active targeting molecules in the particle surface and reduction of toxicity of the entrapped drug [18,19]. LCMs are effectively tested as drug delivery systems for different poorly water-soluble drugs, including tamoxifen [20], paclitaxel [20], vitamin K3 [21], camptothecin [22], norfloxacin [23] and nifedipine [24]. Further polymer-lipid hybrid nanoparticles have been developed to carry and deliver biological drugs, including insulin [25], calcitonin [26], ovalbumin as an oral vaccine model [27] and pEGFP-N2 plasmid DNA as gene therapy for breast cancer [28].

The aim of this investigation was to develop LCMs as drug delivery systems, using fluorescent dye rhodamine 123 (Rho) and human insulin (Ins) as models for poorly water-soluble and biological drugs, respectively, and then inkjet print these drug-loaded LCMs on mucoadhesive films as a final buccal dosage form. This novel formulation resulted in an enhanced permeation of both types of molecules using an ex vivo buccal permeation model, compared to LCMs in suspension and free molecules in solution as controls. Physical and chemical properties of both types of nanoparticles, in vitro release of each molecule from LCMs and mechanical and mucoadhesive properties of films previous and after printing process were also determined.

## 2. Materials and Methods

### 2.1. Materials

Gelucire 44/14^®^ was purchased from Gattefossé (Saint-Priest, France). Tween 20^®^, Span 80^®^, Rho (MW = 380.828 g/mol), human Ins (MW = 5807.629 g/mol), trifluoroacetic acid (TFA), phosphate buffered saline (PBS) tablets pH 7.4, mucin from porcine stomach and membrane dialysis bag MWCO 10,000 kDa were acquired from Sigma-Aldrich (St. Louis, MO, USA). Ethanol, hydrochloric acid, glycerol 85%, and chromatographic grade acetonitrile (ACN) and water were obtained from Merck (Darmstadt, Germany). Hydroxypropyl methylcellulose (HPMC) K100 and ethylcelloluse NF (EC) Standard 10 were purchased from BASF (Ludwigshafen am Rhein, Germany). Triethyl citrate (TEC) was acquired from AK Scientific Inc. (Union City, CA, USA). Vivaspin^®^ 6 100 kDa ultracentrifuge tubes were obtained from Sartorius (Göttingen, Germany). Scissors and tissue slicer carbon steel blades were acquired from Fisher Scientific (Waltham, MA, USA).

### 2.2. Synthesis of Rhodamine 123- and Insulin-Loaded Lipid-Core Micelles

LCMs were prepared by a low-energy hot emulsification process, described by Fritz et al. [24] with slight modifications (Supplementary Materials Figure S1). For this, 1.2 g of Gelucire 44/14^®^, 187.7 mg of Tween 20^®^ and 52.3 mg of Span 80^®^ were used. All chemicals were mixed in a flask placed in a warm water bath at 70 °C under mild stirring for 10 min. For Rho-loaded particles, 0.5 mg of Rho (equivalent to 1.31 μmol) was added to the lipids at the beginning of the heating process, and for Ins-loaded LCMs, a solution containing 1.5 mg of Ins (equivalent to 0.26 μmol) dissolved in 0.75 mL of hydrochloric acid 0.01 N was incorporated after 10 min of stirring, to prevent Ins temperature-induced degradation. After drug incorporation, 2.56 mL of hot milliQ water was added and kept under mild stirring for another 2 min. The flask was removed from the water bath, and 20 mL of cold milliQ water was then rapidly added and stirred for further 5 min. Resulting suspensions were centrifuged at 4000× *g* for 20 min, using Vivaspin^®^ 6, and final supernatants were collected.

### 2.3. Characterization of Rhodamine 123- and Insulin-Loaded Lipid-Core Micelles

Hydrodynamic diameter (HD) and polydispersity index (PdI) measurements were performed by dynamic light scattering using a Zetasizer Nano ZS (Malvern Panalytical Ltd., Malvern, UK) and measurements of 1:10 dilutions of each suspension of LCMs were carried out in triplicate with a detection angle of 173°, at 25 °C in disposable cuvettes. Zeta potential (ZP) measurements were performed by laser Doppler electrophoresis, using Zetasizer Nano ZS and measurements of 1:10 dilutions of each suspension of LCMs were also characterized in triplicate at 25 °C in capillary electrophoresis cells. The particle shape and size of each loaded LCMs were determined in a scanning-transmission electron microscope (STEM) Inspect model F50 (FEI Company, Hillsboro, OR, USA). For this, copper electron microscopy grids were loaded with samples for 2 min, then washed twice with milliQ water for 1 min each, and finally stained with 1% stock solution of phosphotungstic acid for another 2 min.

Entrapment efficiency (EE) and drug loading (DL) of Rho and Ins were determined by an indirect method of quantitation of non-entrapped Rho and Ins. Ultrafiltrates of Rho-loaded LCMs were collected and non-entrapped Rho was measured by Agilent Cary Eclipse fluorescence spectrophotometer (Santa Clara, CA, USA) with emission and excitation wavelengths of 505 and 529 nm, respectively. For non-entrapped Ins quantitation, ThermoFisher Scientific UltiMate 3000 ultra-high performance liquid chromatography (UHPLC) (Waltham, MA, USA) coupled with a UV detector at 215 nm and Brownlee SPP Peptide C_18_ reserved-phase column (2.7 μm, 4.6 mm ID × 100 mm) were used. The mobile phase was composed of a mixture of water:ACN (68:32) and 0.1% of TFA at 1 mL/min and the total time of each analysis was 5 min, with an injection volume of 20 μL. The EE and DL were calculated using Equations (1) and (2), respectively:

(1) $$EE=\;\frac{m_{i}-m_{uf}}{m_{i}}\;\times \;100\;$$

(2) $$DL=\;\frac{m_{i}-m_{uf}}{m_{s}}\;\times \;100$$

where *m_i_* is the initial amount of Rho or Ins added, *m_uf_* is the amount of non-entrapped Rho or Ins after the determination in ultrafiltrates, and *m_s_* is the total amount of solids added to the formulation. To quantify the amount of Rho and Ins, calibration curves in milliQ water of each molecule were prepared.

### 2.4. In Vitro Release Study and Kinetic Modeling of Rhodamine 123- and Insulin-Loaded Lipid-Core Micelles

A total of 4 mL of each Rho- and Ins-loaded LCMs in suspension were transferred to dialysis bags, sealed and suspended in 30 mL of PBS pH 7.4, in triplicate, under mild stirring at 37 °C. In total, 1 mL of each sample was withdrawn at scheduled intervals of 0.25, 0.5, 0.75, 1, 1.5, 2 and 24 h, and replaced with an equivalent volume of fresh PBS. Rho and Ins contents were determined by fluorescence spectroscopy and UHPLC, respectively, as described in Section 2.3. To determine the release mechanism of each drug from LCMs, linear (zero and first orders), and nonlinear regression (Higuchi and Korsmeyer-Peppas) kinetic models were assessed for data fitting and compared based on adjusted coefficient of determination (R^2^) values. For this purpose, the DDSolver program developed for Microsoft Excel was used [29].

### 2.5. Elaboration of Rhodamine 123- and Insulin-Loaded Lipid-Core Micelles Mucoadhesive Films

HPMC K100 was used as a polymer film substrate for the elaboration of buccal mucoadhesive films by solvent casting [30]. A total of 4 g of polymer was heated and stirred with 130 mL of milliQ water, followed by the addition of 2 g of glycerol and 70 mL of cold milliQ water. The final mixture was stored at 4 °C for 24 h, and then 25 g of the solution was portioned into glass plates and dried for 72 h. Further, EC films were elaborated as a control for mucoadhesion experiments. For this purpose, 6 g of polymer were heated and stirred with 50 mL of ethanol, and 0.6 g of TEC was added to the mixture. Then, the solution was cooled at room temperature and ethanol was added to complete 100 mL of the final solution, and then 25 g of the mixture was portioned into glass plates and dried for 48 h.

Rho- and Ins-loaded LCMs mucoadhesive films were obtained by thermal inkjet printing LCM suspensions, using a Hewlett Packard HP Deskjet 1000 printer as reported by Montenegro et al. [31]. Cartridges were loaded with a mixture of 1 mL of Rho- or Ins-loaded LCMs in suspension and 0.45 mL of glycerol, to emulate conventional ink viscosity, for the printing process. After printing, Rho- and Ins-LCMs mucoadhesive films were cut into 1.44 cm^2^ fragments and stored from light until further studies. The amount of Rho or Ins loaded on the surface of films was determined by dissolving in triplicate each film in 4 mL of phosphate buffer pH 6.8 and measured by fluorescence spectroscopy and UHPLC, respectively, as described in Section 2.3.

### 2.6. Characterization of Hydroxypropyl Methylcellulose Mucoadhesive Films

Mechanical properties, mucoadhesion and scanning electron microscopy were conducted for the characterization of HPMC K100 mucoadhesive films before and after the printing process [30]. Insulin-loaded LCMs were used as samples for the printing process of mucoadhesive films for these experiments.

#### 2.6.1. Mechanical Properties

The determination of mechanical properties was performed using a TA1 texture analyzer (Lloyd Instruments Ltd., Bognor Regis, UK) equipped with a 50-N load cell and Nexygen Plus 3 software. Each sample was cut in strips (6 cm × 1 cm) and held between clamps, attached to the equipment, yielding 4 cm^2^ as an effective testing area. The upper clamp exerted a stretching movement to the upper end of each film sample at a rate of 0.5 mm/s until rupture. Measurements were performed in quadruplicate. Furthermore, the thickness of each sample was tested using an electronic micrometer IP54 Mitutoyo (Kawasaki, Japan) with a measurement range from 0 to 25 mm (±0.001 mm precision) and taken at five random positions of each film.

Data were processed using stress versus strain plots. The tensile strength (*TS*) and elongation at break (*EB*) parameters were obtained from the peak stress and the maximum strain, respectively, and calculated using Equations (3) and (4):

(3) $$TS=\;\frac{Peak\;stress}{Cross-sectional\;area\;of\;the\;sample}$$

(4) $$EB=\;\frac{Increase\;in\;length\;at\;break}{Original\;film\;length}\;\times \;100$$

Moreover, the resistance of the sample to be deformed elastically after stress was measured by the elastic or Young’s modulus (EM), defined as the slope of the stress versus strain plot at the initial elastic deformation region.

#### 2.6.2. Mucoadhesive Properties

The determination of mucoadhesion was obtained using the previously described TA1 texture analyzer. Each sample was cut in squares (4 cm × 4 cm) and held horizontally, and 70 μL of freshly made 2% *w*/*v* mucin solution was placed on top of each film, as model mucus mimicking the average saliva thickness. The mucoadhesion assay was conducted by contacting an 11 mm diameter stainless-steel cylindrical probe attached to the mobile arm of the texture analyzer. The probe was brought in contact with the sample and mucin solution with a force of 0.5 mN for 30 s and then withdrawn at a rate of 0.5 mm/s. Parameters of detachment force (DF) and work of adhesion (WoA) were obtained from the peak and area under the curve in the force versus distance profile, respectively. Four independent measurements were performed, and nonprinted EC films were used as control.

#### 2.6.3. Scanning Electron Microscopy

Micrographs of the surface of films were obtained using a scanning transmission electron microscope in its scanning electron microscopy (SEM) mode (Inspect F50, FEI, Hillsboro, OR, USA). The samples were cut into 2 cm × 1 cm pieces and immobilized on aluminum stubs using double-sided adhesive conductive carbon tape. All samples were then coated with gold to a thickness of 10 to 15 nm in a high vacuum evaporator and analyzed using a 5-kV accelerating electron beam.

### 2.7. Ex Vivo Permeation of Rhodamine 123- and Insulin-Loaded Lipid-Core Micelles Mucoadhesive Films

Porcine cheeks were collected from healthy animals from a slaughterhouse in Santiago, Chile. Mucosal epithelia from cheeks were carefully separated from overlying skin and underlying connective and muscular tissues using a surgical scissor and tissue slicer carbon steel blades. Buccal epithelia were cut into approximately 2 cm^2^ surface and equilibrated for 30 min in phosphate buffer pH 6.8, mimicking the saliva, before mounting on Teledyne Hanson Research Phoenix dry heat automated vertical Franz diffusion cells (Chatsworth, CA, USA). The total volume of buffer in the receptor chamber of each diffusion cell was 10 mL at 37 °C and the LCMs-loaded mucoadhesive film was directly loaded on the mucosal epithelium by the printed side, in the donor compartment with 1 mL of buffer. In total, 400 μL of the sample of each diffusion cell were withdrawn every 6 min from 6 to 60 min and the same volume of fresh buffer was replaced. Measurements were conducted as described in Section 2.3 for each molecule. As controls, 1 mL of LCMs in suspension and free molecules in solution were loaded in each diffusion cell at the same experimental conditions. To determine the movement of LCMs across the excised porcine buccal mucosa, the steady-state diffusive flux (*J_ss_*) was determined by the slope of the cumulative concentration in the receiver compartment along the time, and therefore, apparent permeability coefficient (*P_app_*) was calculated using the Equation (5) [32]:

(5) $$P_{app}=\frac{J_{ss}\;\times \;V_{r}}{A\;\times \;C_{0}}$$

where *V_r_* is the volume of the receptor chamber, *A* is the surface cross-sectional area of the buccal epithelium and *C*_0_ is the initial concentration of the compound of the donor chamber.

### 2.8. Statistical and Similarity Analysis

Data analysis was conducted using Minitab 17 (Minitab Inc., State College, PA, USA). The data are represented as the average and the error bars show the standard deviation. Student *t*-test and one-way analysis of variance (ANOVA) with Tukey’s multiple comparison tests were used for each experiment with two or three comparison groups, respectively, to test for significance (*p* < 0.05). Furthermore, a similarity test was performed for assessing the differences among release and permeation curves, using the similarity factor *f_2_* defined as the logarithmic reciprocal square root transformation of the sum of squared error [33], and was calculated according to the Equation (6):

(6) $$f_{2}=50\log\left({\left[1+\frac{1}{n}\sum_{t=1}^{n}{\left(R_{t}-T_{t}\right)}^{2}\right]}^{-0.5}\;\times \;100\right)\;$$

where *n* is the total number of sample points, *R_t_* is the percentage of the released or permeated drug at *t* time of one curve, and *T_t_* is the percentage of the released or permeated drug at *t* time of another curve. In that sense, *f*_2_ values greater than 50 mean that both compared curves have a mean difference not greater than 10% at the sample time point, indicating that both profiles are similar to each other.

## 3. Results

### 3.1. Synthesis and Physical-Chemical Characterization of Rhodamine 123- and Insulin-Loaded Lipid-Core Micelles

HD, PdI and ZP values for Rho- and Ins-loaded LCMs are shown in Table 1. Greater HD values were obtained for Rho-loaded LCMs (26.2 ± 2.5 nm) in comparison with Ins-loaded particles (16.6 ± 1.0 nm) after three independent batches. STEM micrographs confirmed that Rho-loaded LCMs show a bigger particle size rather than Ins-loaded particles (Figure S2A,B, respectively). PdI values of both types of LCMs were similar and demonstrated that low-energy hot emulsification process yields narrow-sized dispersions (Rho-loaded LCMs = 0.251 ± 0.114, and Ins-loaded LCMs = 0.202 ± 0.049). Rho-loaded LCMs showed mildly negative ZP values (−12.4 ± 3.6 mV) in comparison with more neutral charged Ins-loaded LCMs (−2.6 ± 1.1 mV). According to the EE values, Rho (98.3 ± 0.8%) and Ins (94.3 ± 3.8%) were efficiently trapped in LCMs with high association. Furthermore, with the amount of Rho and Ins used during synthesis, DL values were 0.29 ± 0.03% and 0.03 ± 0.00%, respectively.

### 3.2. In Vitro Release Profiles and Kinetic Modeling for Rhodamine 123- and Insulin-Loaded Lipid-Core Micelles

**Figure 1:**
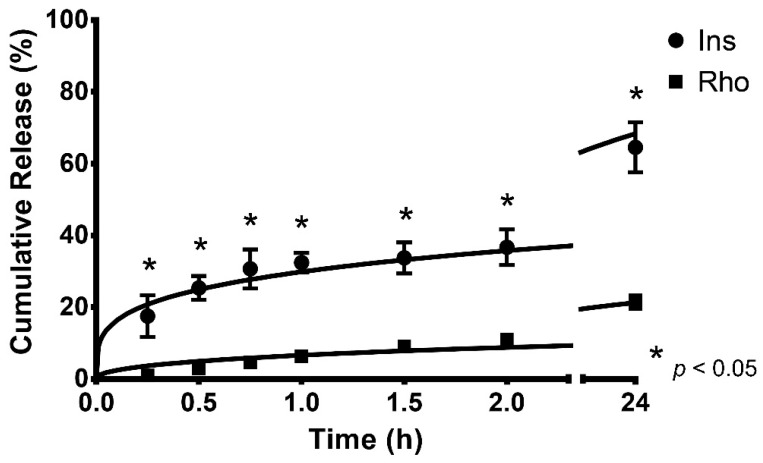
Cumulative in vitro release profiles and kinetic modeling of rhodamine 123 (Rho) and insulin (Ins)-loaded lipid-core micelles, performed at 37 °C, in simulated physiological conditions with PBS pH 7.4. Values are represented as mean ± standard deviation of three different batches (*n* = 3). * indicates statistical differences at the same comparison level by *t*-test (*p* < 0.05).

The release profiles of Rho- and Ins-loaded LCMs are shown in Figure 1 (Table S1). During the first 15 min of the experiment, the cumulative released amount of Ins (17.5 ± 5.8%) was significantly higher than Rho (1.0 ± 0.5%), i.e., approximately 17.5 times greater release (*p* < 0.05). After 24 h, the cumulative released Ins was approximately 3 times higher (64.6 ± 7.0%) than released Rho (21.4 ± 1.8%) (*p* < 0.05).

The kinetic modeling parameters and the adjusted R^2^ values for all release profiles are shown in Table 2. The release profiles of Rho and Ins from LCMs are better fitted by the Korsmeyer–Peppas model (*R*^2^ = 0.969 ± 0.012 and *R*^2^ = 0.989 ± 0.007, respectively). The similarity factor of both profiles was calculated (*f*_2_ = 21.6) resulting in evidently different profiles.

### 3.3. Mechanical and Mucoadhesive Properties of Non-Loaded and Loaded Mucoadhesive Films

The parameters of the mechanical properties of HPMC mucoadhesive films before and after the loading process of LCMS by inkjet printing are shown in Table 3. As expected, there are no significant differences in the thickness of nonloaded and loaded films (78.70 ± 10.30 and 79.45 ± 11.39 μm, respectively). Further, the loading of LCMs on the surface of HPMC films through inkjet printing has no significant effect on the mechanical properties, i.e., TS (8.23 ± 0.96 versus 7.45 ± 0.42 MPa, respectively), EB (73.63 ± 8.28 versus 77.23 ± 8.80%, respectively) and EM (4.40 ± 0.98 versus 4.37 ± 0.68 MPa, respectively).

**Figure 2:**
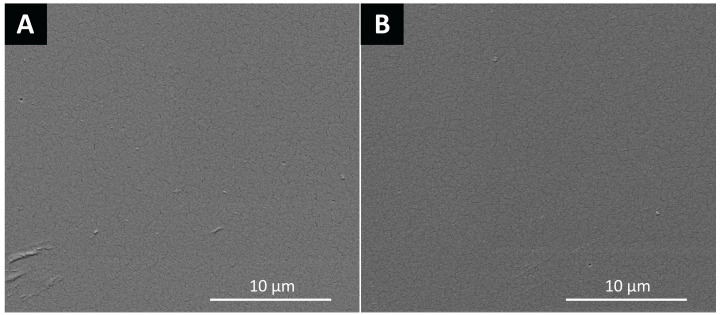
Scanning electron microscopy (SEM) micrographs of the surface of: (**A**) nonloaded and (**B**) loaded hydroxypropyl methylcellulose films.

Moreover, SEM micrographs were obtained and revealed no physical changes on the surface of HPMC films after the printing process (Figure 2).

On the other hand, the mucoadhesive parameters are shown in Table 4. In terms of DF, significant differences were observed among all samples, eliciting a diminished mucoadhesion after the printing process (19.41 ± 4.70 mN) compared to nonloaded films (30.86 ± 2.72 mN), but an enhanced mucoadhesion effect related to EC films as a control (5.27 ± 1.10 mN). A similar but not significant trend was observed regarding to WoA, with nonloaded HPMC films requiring a higher work to separate the sample of the surface (32.32 ± 9.53 mN/mm) rather than loaded films (21.92 ± 10.78 mN/mm) and EC samples (13.95 ± 5.42 mN/mm).

### 3.4. Ex Vivo Permeation for Rhodamine 123- and Insulin-Loaded Lipid-Core Micelles Using Mucoadhesive Films

**Figure 3:**
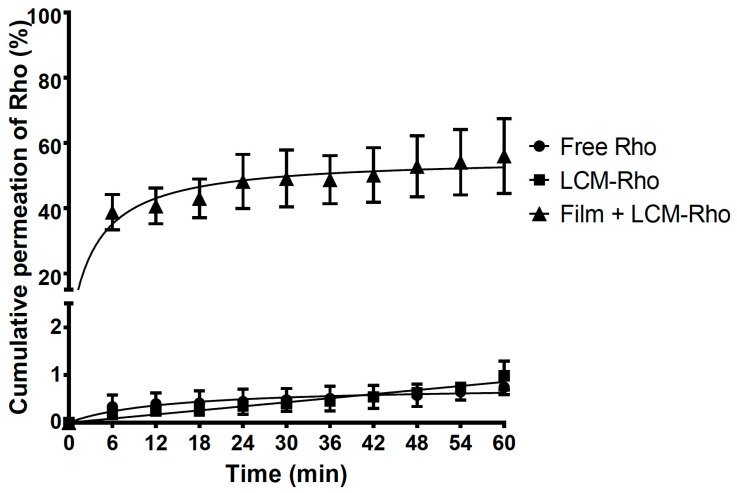
Cumulative ex vivo permeation profiles of rhodamine 123 (Rho) in solution, loaded in lipid-core micelles (LCMs) and in LCMs-loaded on mucoadhesive films through excised porcine buccal epithelium, with phosphate buffer pH 6.8. Values are represented as mean ± standard deviation of six different batches (*n* = 6).

As shown in Figure 3 and Table S2, the permeation profile revealed a significantly higher diffusion of Rho-loaded LCMs printed on HPMC mucoadhesive films (38.81 ± 5.40%) after the first 6 min of experiments in comparison with LCMs in suspension (0.23 ± 0.05%) and free Rho in solution (0.24 ± 0.11%) as controls, i.e., almost 170 times higher permeation for LCMs-loaded mucoadhesive films in comparison with others forms tested (*p* < 0.05). Furthermore, after 60 min of the experiment, the cumulative permeated amount elicited by Rho-loaded LCMs mucoadhesive films increased to 56.02 ± 11.48%, compared to 0.99 ± 0.31% for LCMs in suspension and 0.69 ± 0.09% as free Rho in solution (*p* < 0.05). The difference among these profiles was corroborated with similarity factor values for mucoadhesive films versus in suspension and versus in solution (*f*_2_ = 15.9 for both comparisons).

**Figure 4:**
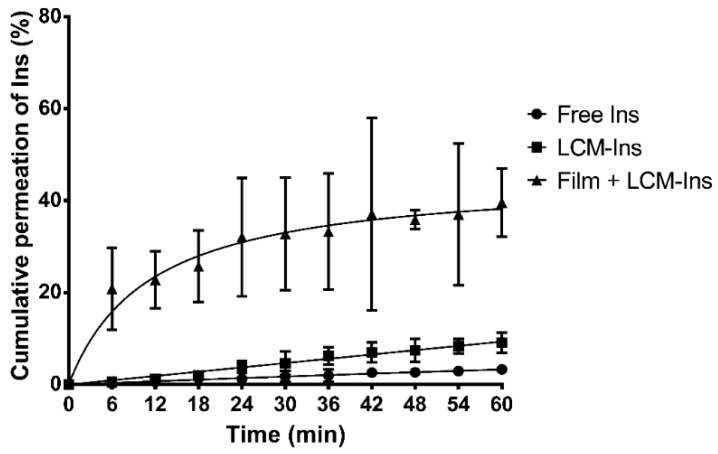
Cumulative ex vivo permeation profiles of insulin (Ins) in solution, loaded in lipid-core micelles (LCMs) and in LCMs-loaded on mucoadhesive films through excised porcine buccal epithelium, with phosphate buffer pH 6.8. Values are represented as mean ± standard deviation of five different batches (*n* = 6).

On the other hand, a significant enhancement of permeation rate was also observed in Ins-loaded LCMs mucoadhesive films (Figure 4 and Table S3). The permeation rate of Ins-loaded LCMs printed on mucoadhesive films (20.85 ± 8.89%) is 36 and 104 times greater than LCMs in suspension (0.58 ± 0.36%) and free Ins in solution (0.20 ± 0.14%) as controls at 6 min (*p* < 0.05). After 60 min, the permeation rate of Ins-loaded LCMs mucoadhesive films increased to 39.59 ± 7.37%, compared to 9.11 ± 2.15% and 3.28 ± 0.69% for LCMs in suspension and free Ins in solution, respectively (*p* < 0.05). The difference among mucoadhesive films versus LCMs in suspension and Ins in solution profiles was also corroborated with *f*_2_ values (28.5 and 25.9, respectively).

Furthermore, diffusion and permeability parameters are shown in Table 5. The *J_ss_* value for Rho-loaded LCMs mucoadhesive films was significantly higher (17.20 ± 3.18 × 10^−3^ ug/cm^2^min) in comparison with LCMs (0.24 ± 0.04 × 10^−3^ ug/cm^2^min) and Rho as free in solution (0.29 ± 0.04 × 10^−3^ ug/cm^2^min) and therefore, elicited a higher flux of particles after reaching the steady-state (*p* < 0.05). Indeed, when using mucoadhesive films, a significantly greater *P_app_* value (2.60 ± 0.48 cm/min) was obtained rather than in LCMs in suspension (0.04 ± 0.01 cm/min) and as free molecules (0.04 ± 0.01 cm/min) (*p* < 0.05). Similar behavior is observed for Ins, leading to greater diffusive flux using LCMs-loaded mucoadhesive films (0.40 ± 0.29 ug/cm^2^min) versus in suspension (0.15 ± 0.02 ug/cm^2^min) or as free Ins (0.05 ± 0.01 ug/cm^2^min). Additionally, significantly higher *P_app_* value was estimated for mucoadhesive films (1.24 ± 0.89 cm/min) rather than LCMs in suspension (0.46 ± 0.08 cm/min) and Ins in solution as controls (0.17 ± 0.04 cm/min).

## 4. Discussion

### 4.1. Physical-Chemical Characterization of Lipid-Core Micelles

The current work is based in assessing Rho and Ins as models for poorly water-soluble and biological drugs, respectively, due to their physical-chemical properties (calculated log P of Rho = 1.5 and slightly to poorly soluble in water [34,35]; water solubility of Ins at pH 7 = 4 mg/mL [36]). In this sense, the higher size elicited by Rho-loaded LCMs could be related to the total amount of Rho used during synthesis rather than the total quantity of Ins employed. The amounts have been selected as proof of concept for the association of both molecules to LCMs. In a similar study, nifedipine-loaded LCMs showed an average diameter of 11.5 ± 2.0 nm [24], demonstrating the feasibility of the low-energy hot emulsification method for achieving small nano-sized lipid particles. A smaller size is a desirable factor and is relevant to improve several biological effects of nanoparticles, including internalization rate, circulation half-lives, penetration through vasculature and reticuloendothelial system uptake [37]. In terms of internalization rate, the small-sized nanoparticles are transported through the buccal epithelium by two different mechanisms: the intracellular or transcellular route, where the system can pass across the cells; and the intercellular or paracellular pathway, where the particle may pass through the spaces between the epithelial cells. Nevertheless, the latter mechanism is the main pathway of transport for small-sized particles across the buccal epithelium [38].

As observed with PdI values, narrow-sized LCMs were obtained. As such, the low-energy hot emulsification process shows several advantages in comparison with traditional high-energy hot and cold high-pressure and high shear homogenization methods, including greater efficiency, lower time and energy consumption, and yielding of small and monodispersed particles [39]. Therefore, the current process is a suitable method for the synthesis of lipid nanoparticles.

According to an online protein calculator [40], the predicted net charge for Ins in acidic conditions has a positive value (from +5.0 at pH 1.0 to +0.8 at pH 5.0), due to the presence of basic amino acid residues, such as histidine, lysine and arginine. Then, the almost-neutral ZP value of Ins-loaded LCMs is due to the interaction between positively charged Ins with the negative constituents of LCMs, specifically, to the free hydroxyl groups derived from PEG residues in the Gelucire 44/14^®^ matrix. Surface charge is an important parameter related to the short- and long-term stability of colloidal dispersions. In that sense, high net ZP values, i.e., either ≥30 mV or ≤−30 mV, are desirable to ensure a monodispersed and pharmaceutically stable dispersion without particle aggregation [41]. The prevention of aggregation is associated with the predominant repulsive forces between adjacent, similarly charged dispersed particles, in a phenomenon so-called electrostatic stabilization [42]. Nonetheless, for polymeric colloidal nanoparticles, including LCMs, there is another type of stabilizing pathway, known as steric stabilization. The steric stabilization is related to the use of high molecular weight hydrophilic polymers and achieved by attaching those molecules to the surface of the particles [43]. Thus, hydrophilic PEG fragments of Gelucire 44/14^®^ may contribute to the steric barrier by extension of their structure into the dispersion medium, in this case, water. Furthermore, nanoparticles with a more neutral surface charge may elicit some advantages, e.g., avoiding macrophage uptake and therefore, increasing the bloodstream circulation time. Indeed, positively charged nanoparticles may trigger the adsorption of negatively charged plasmatic proteins, activating the reticuloendothelial system uptake and impairing the half-life of drug delivery systems [44]. Likewise, there is evidence that negatively charged nanoparticles may potentially bind to positively charged sites on the macrophage surface and then, be recognized and internalized by several surface receptors [45].

Different types of plasmid DNA nanoparticles for mitochondrial-targeting gene therapy yielded lower EE for Rho, ranging from 74% to 81% [46]. On the other hand, other types of lipid nanoparticles have also elicited minor EE of Ins, including liposomes (29% to 42%) [46,47], and lipid hybrid nanocarrier made from PEG and poly(lactic-co-glycolic) acid (PLGA) (20% to 50%) [48]. High entrapment values of both molecules obtained in this study might be related to the constituents of LCMs. While Rho molecules are mainly trapped into the lipid core matrix, due to their lipophilicity, Ins may be mostly confined on the LCM surface, interacting with PEG units. Hydrophilic PEG chains may produce an entangled network around Ins molecules by hydrophobic interactions and additionally, hydrogen bonds are concurrently formed with the surrounding water molecules [49]. Therefore, small-sized, monodispersed and mildly negative-charged LCMs obtained by the low-energy hot emulsification process can be suitable as drug delivery systems for poorly water-soluble and biological drugs, such as Rho and Ins, respectively.

### 4.2. In Vitro Release Profiles of Rhodamine 123 and Insulin

The release of Rho and Ins from LCMs might be related to changes in the transition state of LCMs induced by temperature [50]. In that sense, physical–chemical changes in lipid colloidal systems may be observed, related to the movement from a more compact to a more-lax and fluid state of lipid constituents and therefore, trigger the release of loaded molecules from the lipid matrix. In a similar study using peptide-derivatized PLGA nanoparticles for central nervous system targeting, the cumulative released amount of Rho after 24 h was approximately 35% at 37 °C [51]. The difference may be due to the biphasic release kinetics obtained from PLGA nanoparticles, i.e., a burst effect was observed during the initial hours of experiment, related to loosely bound Rho molecules adsorbed onto the PLGA nanoparticle surface. This burst effect is not prominently observed for Rho-loaded LCMs and could be associated with the presence of tighter interactions between Rho and lipid constituents forming a Rho-enriched core, unlike loosely bound Rho on to the PLGA nanoparticle surface.

Contrastingly, with Rho-loaded LCMs, Ins-loaded particles did elicit a burst release effect during the first 2 h of experiments, eliciting a significantly higher cumulative release throughout the experiment. Ins molecules, as described in Section 4.1, may be entangled with hydrophilic PEG moieties of Gelucire 44/14^®^, in the outer part of LCMs and therefore, the interactions could be weaker than Rho. Furthermore, the adsorption of water molecules to the PEG moieties is reported, leading to changes from the polymer crystalline structure to a less organized conformation, and consequently, promoting the release of the entrapped Ins [50]. Park et al. also demonstrated a rapid initial release of Ins during the first 12 h, and then a consistent behavior reaching approximately 60% of cumulative released amount after 24 h, in non-PEGylated cationic liposomes [52]. Therefore, lipid nanoparticles, including LCMs, are suitable for Ins delivery, eliciting an initial burst release followed by a more sustained and controlled release.

The release of Rho and Ins are best adjusted to the Korsmeyer–Peppas kinetic model, according to the R^2^ value. Briefly, this kinetic model explains drug release by two different mechanisms: diffusion and case II transport [53]. While the former pathway is governed by Fick’s diffusion law, i.e., the movement of particles depend on the concentration gradient; the latter is an anomalous non-Fickian process, where the diffusing penetrant can cause a deformation and relaxation of the system inducing erosion, swelling and stress in the material and promoting the diffusion process [54]. In this model for spherical particles, if the release coefficient *n* has a value of 0.43 or less (*n* ≤ 0.43), the drug transport pathway is governed by the Fick’s diffusion law; if the *n* exponent is between 0.43 and 0.89 (0.43 < *n* < 0.89), the transport profile shows an anomalous non-Fickian behavior; if *n* equals 0.89 (*n* = 0.89), the non-Fickian transport is related with the case II mechanism; and if the exponent is greater than 0.89 (*n* > 0.89), the drug transport follows a non-Fickian diffusion process known as super case II transport [55,56]. Then, with the calculated *n* value (0.401 ± 0.150), the release of Rho from LCMs is attributed to a diffusion mechanism. Moreover, the release of Ins is also dependent on diffusion governed by Fick’s law (*n* = 0.261 ± 0.014). This might be due to the fact that higher temperatures may induce water adsorption through the hydrophilic constituents of Gelucire 44/14^®^ and then, go through the lipid core, forcing the diffusion of molecules to the medium. As discussed in Section 4.1, Ins molecules are entangled at the surface of LCMs and therefore, prone to the contact with water moieties forcing a rapid release to the medium, whereas the rate of Rho release is slower due to the high entrapment into the core of LCMs. Although the *f*_2_ value indicates that both release profiles are different, the underlying mechanism might be similar.

In polymeric nanoparticles, including LCMs, the mechanism of drug release is related to the drug itself, polymer, ratio of composition, physical and chemical interaction among components and the elaboration process [57]. In that sense, Gelucire 44/14^®^ acts as a water-dispersible surfactant and enhances the wettability by reducing the surface tension between LCMs and the medium, minimizing the aggregation and promoting the dissolution of poorly water-soluble drugs [58]. This polymer has been reported as a material for the elaboration of modified release drug delivery systems, due to the hydrophobicity of the composition of this polymer, based on a mixture of PEG glycerides and esters [59]. Furthermore, interfacial distribution and transfer may also affect the drug dissolution and release to the medium, i.e., the type of the constituents of the nanoparticles will determine the affinity, and therefore, the partition of the drug within the nanoparticle or the aqueous phase. In this sense, the release of hydrophilic drugs, such as Ins, from micelles is a rapid process, due to the fast removal of the drug from the medium that creates an augmented aqueous concentration difference and thus enhancing the migration of molecules. Nonetheless, for poorly water-soluble drugs, such as Rho, the release from micelles is slower, due to high affinity for the lipid constituents and therefore, the migration and transfer of drugs to water are less favored [60]. In this sense, LCMs made of Gelucire 44/14^®^ may serve as drug delivery systems when a modified or controlled release of the entrapped poorly water-soluble drug is desired. Subsequently, the release of Rho and Ins from LCMs is mediated by a diffusive-controlled mechanism. Moreover, a higher cumulative released amount of Ins rather than Rho may be a consequence of their distinct water-solubility and then, affect the migration and mass transfer to the medium.

### 4.3. Mechanical and Mucoadhesive Properties of Films

Inkjet printing was previously reported as a method for loading molecules on polymeric films [30]. This printing method is described as a contactless process able to load functional materials as an ink on a selected surface of a substrate with a desired printing pattern that can be previously designed in a computer [61]. The mechanism is related to the formation of droplets by a heating process that may rapidly rise to 300–400 °C to evaporate the ink solution and create a bubble pushing a small-size droplet of ink out of the nozzle [61]. Main advantages of inkjet printing include the cost-effectiveness of the process, customizable method for loading molecules for different purposes (i.e., the design of personalized dosage forms) and accurate deposition of material at predesigned locations of a substrate; whereas the possibility of nozzle clogging, complex drying behavior and the ink composition remain as the major challenges of this printing process [61,62]. According to printer specifications used in the current investigation, the droplets are uniformly generated with a reported volume of 13.5 pL and a diameter ranging from 20 to 50 μm [62,63]. As reported by Montenegro-Nicolini et al., the inkjet printing process (including the high temperature exposure) has no impact on the structure of printed lysozyme and ribonuclease-A, with minor effects in their enzymatic activity [31].

Furthermore, HPMC is a nonionic cellulose-derived semisynthetic polymer recognized as generally regarded as safe (GRAS) excipient by the FDA. HPMC is commonly used as an emulsifier, thickening and suspending agent in eye drops and controlled-delivery systems in oral products [64]. Particularly, HPMC K100 has been described to have 22% and 8.1% of methoxyl and hydroxypropyl groups, respectively, and a viscosity of 100 cP at 20 °C in a 2% aqueous solution [65].

In terms of mechanical properties, both analyzed films have elicited lower TS values, i.e., the maximum stress that the samples can withstand while being stretched before breaking, compared to reported pure HPMC films [66,67]. This may be related to the incorporation of glycerol as a plasticizer, reducing the intermolecular forces between polymer chains and providing a lamellar structure, leading to a reduction in TS values [30,68]. The printing process has no significant effect on TS; however, there is a relationship between this parameter and the size of loaded-nanoparticles, where smaller particle sizes may not fully occupy the empty spaces of the pores on the surface of the films rather than greater size nanoparticles, resulting in less stress-resistant samples [69]. Moreover, there are no significant differences in the maximum length of deformation of the film while stretching is performed on the samples before breaking; nonetheless, these EB values for both analyzed films are greater than reported HPMC films and may be due to the greater thickness of tested films [30,67,69]. The elongation of both samples can be confirmed by their elastic behavior, eliciting similar EM values, and therefore, lower stiffness compared to several reported HPMC films [30,67,69].

In terms of mucoadhesive parameters, HPMC films have shown higher adhesion properties rather than EC control samples as expected, translating into greater values of DF and WoA. Bagul et al. have also demonstrated that HPMC elicited greater force of adhesion compared to EC [70]. The mucoadhesion elicited by HPMC is related to the polar functional groups, specifically, free and nonsubstituted hydroxyl groups. Those groups can interact with mucins by physical and chemical interactions, including hydrogen bonds [71]. Another key parameter is related to the viscoelastic properties of HPMC. The sol-gel transition or gelation process is an important parameter of fluidity in colloidal systems and is related to the wetting, hydration, and swelling processes of the polymer [72]. A good fluidity is achieved when a liquid-like or sol state is present; however, a gel formation is closely related to the loss of fluidity and increased viscosity [73]. Therefore, as HPMC K100 has a high and low content of methoxyl and hydroxypropyl substitutions, respectively, the polymer elicits a good network entanglement and a fluid liquid-like or weak gel state promoting the adhesion with the mucus. Considering the HPMC samples, the printing process may influence the adhesive properties of the polymer, as shown in DF and WoA values. The printing process disposes ink in an aqueous state, that is absorbed by the film within a few seconds, promoting an initial wetting of the polymer, but not enough to trigger the swelling, and hence, the gelation process [74]. Despite the diminished DF, the loaded HPMC films preserve optimal mucoadhesive behavior.

### 4.4. Ex Vivo Permeation of Rhodamine 123 and Insulin

The enhanced permeation effect observed may be attributed to the mucoadhesive properties of the HPMC as a permeation enhancer. As discussed above, the mucoadhesive behavior of the polymer is related to the interactions between polar groups and mucins, prolonging the residence time of the formulation at the immediate mucosal surface and promoting the diffusion process through the buccal epithelium. Further, the sol-gel transition process due to wetting, hydration and swelling of the polymer, may create a force that pushes the Rho- and Ins-loaded particles through the buccal epithelium [75,76].

Nonetheless, a 1.4 times greater permeation rate trend is observed for Rho-loaded LCMs mucoadhesive films rather than for Ins-loaded LCMs HPMC films after 60 min. This fact may be related to the surface charge observed in both types of LCMs. There is evidence that anionic compounds may increase the energy barrier of the sol-gel transition, i.e., favoring the liquid-like instead of a more viscous state, due to salting in process [77]. Hence, negatively charged Rho-loaded LCMs can stimulate the maintenance of the HPMC sol state in contact with water and consequently, promote the free movement of nanoparticles between the dosage form and the surrounding areas. Nonetheless, more neutral charged Ins-loaded LCMs may not increase the energy barrier of sol-gel transition and a less fluid gel state of HPMC is preserved, which may impair the free movements of nanoparticles on the dosage form and the surrounding areas.

On the other hand, LCMs themselves may also enhance the permeation rate of Rho and Ins compared with free molecules in solution. There is evidence suggesting that several types of fatty acids, including lauric acid (the main constituent of Gelucire 44/14^®^) may act as a buccal permeation enhancer. The mechanism is related to the lipophilicity of fatty acids, promoting interactions with several types of lipids in the intercellular spaces of the buccal epithelium, including ceramides, saturated fatty acids and cholesterol, which play a role as a barrier [78,79]. Furthermore, other constituents of developed LCMs may also serve as buccal permeation enhancers. Surfactants, including different types of Tween^®^, enhance the paracellular permeability of several compounds by perturbation of intercellular lipids and protein domains integrity [80]. PEG is reported as a skin permeation enhancer by altering the solution properties of the intercellular lipids of the subcutaneous tissue and therefore, impairing the barrier function of this layer [81]. Despite the histological differences between the subcutaneous tissue and the buccal epithelium, PEG with a buccal permeation enhancer function by a similar mechanism cannot be excluded. In consequence, LCMs might improve the permeation process of the loaded drug by promoting the interaction with intercellular lipids and the perturbation of the lipid barrier of buccal mucosa, enhancing the paracellular transport. Singularly, a nine times higher permeation rate for Ins-loaded LCMs in suspension is achieved in comparison with Rho-loaded LCMs in suspension (*p* < 0.05). The negatively charged mucins of buccal epithelium are not an impediment for the diffusion and penetration of almost neutral surface charge of Ins-loaded LCMs, contrary to what can be expected by more negative-charged particles, such as Rho-loaded LCMs in suspension, where the electrostatic repulsion forces may impair the diffusion of these particles across the buccal epithelium. The large deviation in permeation results throughout might be due to the intrinsic variability for using ex vivo excised porcine cheeks in permeation experiments rather than well-controlled, but physiologically less representative cell culture models [32]. Furthermore, the greater variability observed for Ins rather than Rho-loaded loaded LCMs mucoadhesive films might be related with the interaction between Ins and the components of the buccal epithelium, increasing the retention of Ins into the mucosal layers and also favoring the degradation of the molecule, due to the presence of extracellular enzymes, such as aminopeptidases [82,83].

Other types of Ins-loaded nanoparticles mucoadhesive films for buccal administration were reported. Eudragit RLPO^®^ alone or combined with HPMC were used as a substrate for the elaboration of buccal mucoadhesive film prototypes and loaded with Ins-coated valine nanoparticles. The results demonstrated a sustained release of Ins through hours, with an approximately 5% of the cumulative amount of Ins released after 30 min from Eugragit RLPO^®^ films, and almost null for polymer-mixed films [84]. This delayed release in comparison with HPMC mucoadhesive films may be attributed with the addition of Eudragit RLPO^®^, a water-insoluble and swellable polymer with quaternary ammonium groups [85]. The positively charged substitution may enhance the interaction with the negative-charged mucins and also modify the release of Ins-coated nanoparticles through hours. In another research, the cumulative permeation of Ins from thiolated chitosan nanoparticles loaded on chitosan mucoadhesive films was not greater than 15% after 30 min of an ex vivo permeation experiment [76]. Chitosan is also a cationic polymer that can interact with negatively charged groups of the buccal epithelium, enhancing the mucoadhesive properties [86]. As discussed above, the swelling of a polymer is an important parameter to promote an effective release of loaded content. Therefore, the high permeation of Ins-loaded particles from HPMC films evinced by the current study in comparison with Eudragit RLPO^®^ or chitosan films may be related to the greater ability of HPMC to adsorb water, stimulating a more fluid state and then enhancing the diffusion of nanoparticles.

Considering the aspects discussed in the current section, using HPMC mucoadhesive films may represent a clear advantage for delivering drugs through buccal administration. An increase in local concentration due to immediate adherence to the buccal mucosa or mucoadhesion, and therefore, fix the substrate to the absorptive epithelium of cheeks, may push the Rho- and Ins-loaded LCMs to pass through the epithelium, and prevent the constant loss of drugs due to salivation and swallowing phenomena. Therefore, LCMs loaded on HPMC mucoadhesive films may constitute a novel buccal dosage form for the administration of poorly water-soluble and biological drugs. Furthermore, this type of buccal drug delivery system may contribute to an increase in patient compliance without disturbing normal functions including talking, eating or drinking, in comparison with other dosage forms such as buccal tablets.

## 5. Conclusions

LCMs-loaded HPMC mucoadhesive films were successfully developed as a novel buccal drug delivery system for Rho and Ins, as models for poorly water-soluble drugs and biologics, respectively. LCMs were small-sized, monodispersed and spherical-shaped nanoparticles, with high entrapment efficiency for both molecules. The release profiles of Rho and Ins from LCMs at 37 °C are governed by Fickian diffusion. Nonetheless, an initial and significant burst release was observed during the first hours of the experiment for Ins-loaded LCMs due to high entrapment on the surface of these nanoparticles, rather than Rho release which is slower due to the association with lipid constituents of the LCMs. The inkjet printing process has no significant effects on the mechanical properties of HPMC films; and maintaining optimal mucoadhesive behavior. HPMC mucoadhesive films have demonstrated an enhanced permeation of Rho- and Ins- through ex vivo porcine buccal epithelium. The mucoadhesive and viscoelastic properties of the polymer may increase the contact time between the film and the absorptive mucosa. Furthermore, lipid constituents of synthesized LCMs may also enhance the permeation of both tested molecules.

## Figures and Tables

**Table 1:** Physical-chemical properties of rhodamine 123 (Rho) and insulin (Ins)-loaded lipid-core micelles (LCMs). Values are presented as mean ± standard deviation (*n* = 3).

LCMs	HD (nm)	PdI	ZP (mV)	EE (%)	DL (%)
Rho	26.2 ± 2.5	0.251 ± 0.114	−12.4 ± 3.6	98.3 ± 0.3	0.29 ± 0.03
Ins	16.6 ± 1.0	0.202 ± 0.049	−2.6 ± 1.1	94.3 ± 3.8	0.03 ± 0.00

HD: hydrodynamic diameter; PdI: polydispersity index; ZP: zeta potential; EE: entrapment efficiency; DL: drug loading.

**Table 2:** Kinetic modeling parameters and adjusted correlation coefficient (*R*^2^) for in vitro release profiles of rhodamine 123 (Rho) and insulin (Ins)-loaded lipid-core micelles, performed at 37 °C. Values are presented as mean ± standard deviation (*n* = 3).

Kinetic Model	Correlation Coefficient	Rho	Ins
Zero Order Q = kt + b	$R^{2}$	0.896 ± 0.025	0.939 ± 0.013
	k	0.447 ± 0.065	1.495 ± 0.203
First Order $Q=100\;\times \;\left(1-e^{-kt}\right)$	$R^{2}$	0.909 ± 0.026	0.971 ± 0.003
	k	0.005 ± 0.001	0.058 ± 0.031
Higuchi Q=kt12	$R^{2}$	0.957 ± 0.016	0.979 ± 0.003
	k	3.567 ± 0.495	12.246 ± 1.638
Korsmeyer-Peppas $Q=kt^{n}$	$R^{2}$	0.969 ± 0.012	0.989 ± 0.007
	k	5.332 ± 1.417	29.855 ± 4.167
	n	0.401 ± 0.150	0.261 ± 0.014

**Table 3:** Parameters of mechanical properties for nonloaded (NL-HPMC) and loaded (L-HPMC) mucoadhesive films. Values are presented as mean ± standard deviation (*n* = 4).

Films	Thickness (μm)	TS (MPa)	EB (%)	EM (MPa)
NL-HPMC	78.70 ± 10.30	8.23 ± 0.96	73.63 ± 8.28	4.40 ± 0.98
L-HPMC	79.45 ± 11.39	7.45 ± 0.42	77.23 ± 8.80	4.37 ± 0.68

TS: tensile strength; EB: elongation at break; EM: elastic modulus.

**Table 4:** Parameters of mucoadhesion properties for nonloaded (NL-HPMC) and loaded (L-HPMC) mucoadhesive films. Values are presented as mean ± standard deviation (*n* = 4). Different superscript letters in each column indicates statistical differences for each parameter by one-way analysis of variance with Tukey’s multiple comparison tests (*p* < 0.05).

Films	DF (mN)	WoA (mN/mm)
NL-EC	5.27 ± 1.10 ^A^	13.95 ± 5.42 ^A^
NL-HPMC	30.86 ± 2.72 ^B^	32.32 ± 9.53 ^A^
L-HPMC	19.41 ± 4.70 ^C^	21.92 ± 10.78 ^A^

NL-EC: nonprinted ethyl cellulose films (control); DF: detachment force; WoA: work of adhesion.

**Table 5:** Steady-state diffusive flux (*J_ss_*) and apparent permeability coefficient (*P_app_*) parameters for ex vivo permeation profiles of rhodamine 123 and insulin in solution, in lipid-core micelles (LCMs) and LCMs-loaded mucoadhesive films. Different superscript letters (A, B) in the same row and category indicates statistical differences by one-way analysis of variance with Tukey’s multiple comparison tests (*p* < 0.05).

Parameters	Rhodamine 123			Insulin		
	Solution	LCMs	Films	Solution	LCMs	Films
$J_{ss}$ $\left(\mu g/cm^{2}min\right)$	0.00029 ± 0.00004 ^A^	0.00024 ± 0.00004 ^A^	0.01720 ± 0.00318 ^B^	0.05293 ± 0.01245 ^A^	0.14838 ± 0.02679 ^A^	0.39803 ± 0.28664 ^B^
$P_{app}\;\left(cm/min\right)$	0.036 ± 0.006 ^A^	0.044 ± 0.006 ^A^	2.599 ± 0.481 ^B^	0.165 ± 0.039 ^A^	0.462 ± 0.083 ^A^	1.239 ± 0.892 ^B^

## Supplementary Materials

The following are available online at https://www.mdpi.com/1999-4923/12/12/1168/s1, Figure S1. Schematization of the elaboration process of different drug-loaded lipid-core micelles, using the method of low-energy hot emulsification; Figure S2. Scanning transmission electron microscopy (STEM) micrographs of: (A) rhodamine 123-loaded lipid-core micelles, and (B) insulin-loaded lipid-core micelles; Table S1: Cumulative in vitro release values of rhodamine 123 (Rho) and insulin (Ins) from lipid-core micelles, at 37 °C using PBS pH 7.4; Table S2: Cumulative ex vivo permeation values of rhodamine 123 in solution, loaded in lipid-core micelles (LCMs) and in LCMs-loaded on mucoadhesive films through excised porcine buccal epithelium, with phosphate buffer pH 6.8; Table S3: Cumulative ex vivo permeation values of insulin in solution, loaded in lipid-core micelles (LCMs) and in LCMs-loaded on mucoadhesive films through excised porcine buccal epithelium, with phosphate buffer pH 6.8.
