# Supplementary material for: Drug-Loaded Lipid-Core Micelles in Mucoadhesive Films as a Novel Dosage Form for Buccal Administration of Poorly Water-Soluble and Biological Drugs
Source: Pharmaceutics. 2020 Nov 30;12(12):1168. doi: 10.3390/pharmaceutics12121168 (PMC7761273; doi:10.3390/pharmaceutics12121168)
Supplement: Supplementary file 1 [file pharmaceutics-12-01168-s001.pdf]

pharmaceutics

Supplementary Material: Drug-Loaded Lipid-Core Micelles in Mucoadhesive Films as a Novel Dosage Form for Buccal Administration of Poorly Water-Soluble and Biological Drugs

Wai-Houng Chou, Ariel Galaz, Miguel O. Jara, Alexander Gamboa, and Javier O. Morales

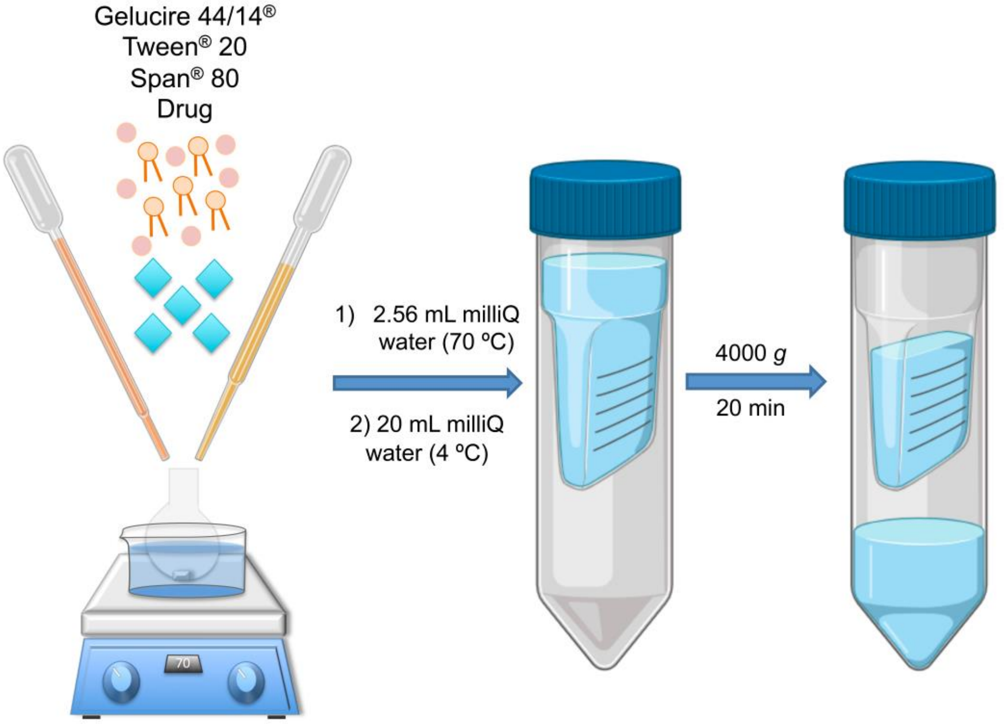

Figure S1. Schematization of the elaboration process of different drug-loaded lipid-core micelles, using the method of low-energy hot emulsification.

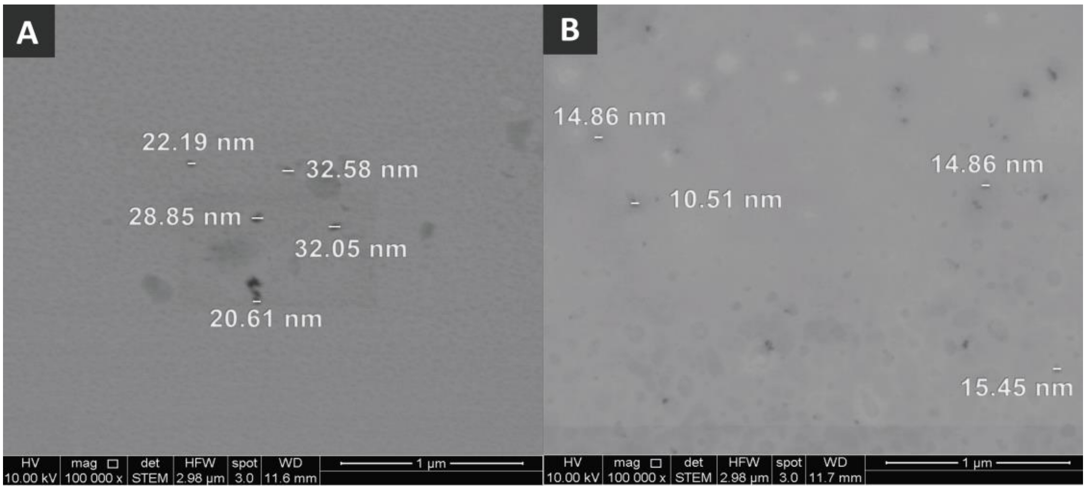

Figure S2. Scanning transmission electron microscopy (STEM) micrographs of: (A) rhodamine 123-loaded lipid-core micelles, and (B) insulin-loaded lipid-core micelles.

Table S1. Cumulative *in vitro* release values of rhodamine 123 (Rho) and insulin (Ins) from lipid-core micelles, at 37 ºC using PBS pH 7.4. Values are represented as mean ± standard deviation of three different batches (n = 3). Different superscript letters in the same row indicates statistical differences by *t*-test ($p < 0.05$).

Time (h)	Rho (%)	Ins (%)
0.25	1.00 ± 0.53^{A}	17.52 ± 5.80^{B}
0.50	2.92 ± 0.85^{A}	25.38 ± 3.30^{B}
0.75	4.72 ± 0.92^{A}	30.69 ± 5.39^{B}
1.00	6.37 ± 1.02^{A}	32.42 ± 2.70^{B}
1.50	9.04 ± 1.18^{A}	33.75 ± 4.30^{B}
2.00	11.00 ± 1.06^{A}	36.71 ± 4.92^{B}
24	21.40 ± 1.83^{A}	64.55 ± 6.96^{B}
48	24.67 ± 3.68^{A}	81.01 ± 13.10^{B}
72	25.88 ± 4.22^{A}	87.85 ± 11.50^{B}

Table S2. Cumulative *ex vivo* permeation values of rhodamine 123 in solution, loaded in lipid-core micelles (LCMs) and in LCMs-loaded on mucoadhesive films through excised porcine buccal epithelium, with phosphate buffer pH 6.8. Values are represented as mean ± standard deviation of six different batches (n = 6). Different superscript letters in the same row indicates statistical differences by one-way ANOVA with Tukey's multiple comparison tests ($p < 0.05$).

Time (min)	Cumulative permeation of rhodamine 123 (%)		
	In solution	LCMs	LCMs-Film
6	0.24 ± 0.11^{A}	0.23 ± 0.05^{A}	38.81 ± 5.40^{B}
12	0.30 ± 0.08^{A}	0.24 ± 0.05^{A}	40.68 ± 5.46^{B}
18	0.31 ± 0.08^{A}	0.30 ± 0.04^{A}	42.99 ± 5.92^{B}
24	0.34 ± 0.11^{A}	0.35 ± 0.03^{A}	48.20 ± 8.27^{B}
30	0.39 ± 0.10^{A}	0.40 ± 0.04^{A}	49.13 ± 8.74^{B}
36	0.41 ± 0.11^{A}	0.45 ± 0.05^{A}	48.75 ± 7.39^{B}
42	0.46 ± 0.13^{A}	0.54 ± 0.05^{A}	50.16 ± 8.38^{B}
48	0.48 ± 0.09^{A}	0.63 ± 0.10^{A}	52.84 ± 9.31^{B}
54	0.58 ± 0.08^{A}	0.72 ± 0.08^{A}	54.10 ± 10.01^{B}
60	0.69 ± 0.09^{A}	0.99 ± 0.31^{A}	56.02 ± 11.48^{B}

Table 3. Cumulative *ex vivo* permeation values of insulin in solution, loaded in lipid-core micelles (LCMs) and in LCMs-loaded on mucoadhesive films through excised porcine buccal epithelium, with phosphate buffer pH 6.8. Values are represented as mean ± standard deviation of five different batches (n = 5). Different superscript letters in the same row indicates statistical differences by one-way ANOVA with Tukey's multiple comparison tests ($p < 0.05$).

Time (min)	Cumulative permeation of insulin (%)		
	**In solution**	**LCMs**	**LCMs-Film**
6	0.20 ± 0.14^{A}	0.58 ± 0.36^{A}	20.85 ± 8.89^{B}
12	0.55 ± 0.48^{A}	1.17 ± 0.63^{A}	24.64 ± 6.85^{B}
18	$1,40 \pm 0.75^{A}$	1.99 ± 0.72^{A}	25.71 ± 7.79^{B}
24	1.25 ± 1.07^{A}	3.80 ± 1.32^{A}	32.09 ± 12.84^{B}
30	1.77 ± 1.22^{A}	4.63 ± 2.55^{A}	32.79 ± 12.23^{B}
36	1.94 ± 1.34^{A}	6.23 ± 1.86^{A}	33.28 ± 12.63^{B}
42	2.59 ± 1.10^{A}	7.00 ± 2.13^{A}	37.08 ± 20.94^{B}
48	2.65 ± 0.82^{A}	7.45 ± 2.47^{B}	35.86 ± 2.04^{C}
54	2.95 ± 1.08^{A}	8.39 ± 1.58^{A}	37.03 ± 15.44^{B}
60	3.28 ± 0.69^{A}	9.11 ± 2.15^{A}	39.59 ± 7.37^{B}
